# Associations between pre-COVID-19 physical activity profiles and mental wellbeing and quality of life during COVID-19 lockdown among adults

**DOI:** 10.1007/s12144-022-03413-3

**Published:** 2022-08-13

**Authors:** Kara Dadswell, Matthew Bourke, Jaimie-Lee Maple, Melinda Craike

**Affiliations:** 1 Institute for Health and Sport, Victoria University, Melbourne, Australia; 2 Mitchell Institute, Victoria University, Melbourne, Australia; 3 Institute for Health and Sport, Victoria University, 8001 Melbourne, VIC P.O Box 14428, Australia

**Keywords:** Physical activity, Mental health, Mental wellbeing, Quality of life, COVID-19, Latent profile analysis

## Abstract

The COVID-19 pandemic has been detrimental to the physical and mental health and wellbeing of people across the globe. Regular physical activity has consistently demonstrated an array of health benefits, but the impact of regular physical activity habits pre-pandemic on health and wellbeing during the pandemic is largely unknown. The purpose of this study was to identify distinct pre-COVID-19 lockdown physical activity profiles [i.e., walking, leisure-time moderate-vigorous physical activity (MVPA), domestic MVPA and muscle strengthening exercise] and assess whether these profiles were associated with mental wellbeing and quality of life during COVID-19 lockdown. A total of 442 adults (Mage = 43.97 ± 13.85; 75.6% female) from Melbourne, Australia completed an online questionnaire measuring pre-COVID-19 physical activity, including walking habits, leisure-time MVPA, domestic MVPA, and muscle strengthening exercise – and completed measures of mental wellbeing and health related quality of life. Latent profile analysis identified five distinct profiles that differed in terms of levels of walking, leisure-time MVPA, domestic MVPA and muscle strengthening exercise. Based on the observed pre-COVID-19 lockdown profiles, it appears that high levels of MVPA and muscle strengthening exercise may serve as a protective factor against the potential negative impact of a global pandemic lockdown on mental wellbeing and quality of life.

## Introduction

The COVID-19 pandemic has affected the physical and mental health and wellbeing of people worldwide (Stockwell et al., 2021; Vindegaard & Benros, 2020). By April 2020, over 100 countries had implemented lockdown measures of varying degrees (Füzéki et al., 2020). Melbourne, Australia was among those experiencing the longest and strictest lockdowns in the world. In essence, lockdowns restricted human movement and interaction as Governments imposed rules to reduce the opportunity for the coronavirus to spread (e.g., parameters on reasons, distance, and time permitted to leave home). A systematic review of the evidence surrounding the mental health consequences of the COVID-19 pandemic reported a decline in mental health among the general population (Vindegaard & Benros, 2020).

The health benefits of regular participation in aerobic physical activity and muscle strengthening exercises are well documented (Czosnek et al., 2019; Warburton & Bredin, 2017). The World Health Organization’s ([WHO], 2020) Physical Activity Guidelines provide minimum recommendations for type (e.g., aerobic, muscle strengthening exercises) and degree (e.g., duration, intensity, frequency) of physical activity for adults to achieve substantial health benefits. These guidelines suggest that adults should accumulate at least 150–300 min of moderate intensity aerobic physical activity, 75–150 min of vigorous intensity aerobic physical activity, or an equivalent combination of both each week, in addition to doing muscle strengthening exercise of at least moderate intensity on two or more days a week. However, one in four adults does not meet these guidelines (WHO, 2020). Although these guidelines are based on overall moderate-to-vigorous physical activity (MVPA), the mental health benefits of MVPA may be domain specific, with leisure-time MVPA being more strongly associated with mental health than other domains, such as work-related physical activity (Craike et al., 2019; White et al., 2017a).

Studies examining the association between physical activity and mental health have historically focused on mental illness (e.g., depression, schizophrenia), rather than constructive mental wellbeing (Tamminen et al., 2020). Positive psychology postulates that mental wellbeing is the product of positive feelings (i.e., hedonia) and effective functioning (i.e., eudaimonia) (Keyes & Annas, 2009), and whilst the absence of mental illness is regularly used as a proxy measure for mental wellbeing, they are distinct constructs (Greenspoon & Saklofske, 2001; Stewart-Brown et al., 2015). Improvements in mental illness are relevant for only a portion of people. In contrast, mental wellbeing is a state that can be achieved by all people, including those experiencing mental illness (Keyes & Annas, 2009). Hence, there is scope to examine the influence of physical activity on mental wellbeing, which is particularly relevant during a pandemic affecting the global population.

Several studies have examined the impact of physical activity on mental wellbeing during COVID-19 lockdown periods. For example, Jacob et al. (2020) explored the relationship between physical activity and mental health in the general UK population during COVID-19 lockdown, finding that greater physical activity throughout lockdown was associated with better mental health and wellbeing. Another study focused on changes in physical activity since lockdown and the association with wellbeing (i.e., anxiety, general mental-health) in previously (pre-COVID-19) active and inactive adults (Lesser & Nienhuis, 2020). Findings determined that previously inactive adults who had become more active or maintained their activity levels during COVID-19 had higher social, emotional, and psychological health and lower levels of generalized anxiety than those who became more inactive. Interestingly, no differences in overall wellbeing were identified in the previously active group between those whose activity levels remained stable or altered (i.e., either increased or decreased). Stable levels of mental health for those who were considered active pre-COVID-19, but had reduced activity during COVID-19, suggests that regular physical activity could be a protective factor for mental wellbeing during challenging times such as a global pandemic. Zimmermann et al. (2020) explored pre-COVID-19 modifiable health-related behaviors as protective factors for psychological health in University students during the early stages of COVID-19 lockdown. None of the pre-COVID-19 health behaviors served as protective factors, however, psychological health data was collected in the early stages of COVID-19 lockdown (April 2020), and therefore it is unclear if longer-term benefits existed.

Although there has been research examining the association between physical activity and mental health *during* COVID-19 lockdowns, these studies have used conventional methods to examine associations (e.g., linear regression), potentially limiting our understanding of the relationship. Variable centered approaches can indicate the independent association between variables, but they are limited in their ability to identify interactive effects between multiple independent variables with an outcome (Muthén & Muthén, 2000). Therefore, variable centered approaches are not well suited to understanding the interaction between distinct physical activity behaviors and their association with outcomes of interest. Researchers may also use cut-points (e.g., MVPA and muscle strengthening exercise according to recommendations) to combine distinct physical activity behaviors, but this is also limited because it does not consider the dose-response relationship between physical activity behaviors and outcomes (Brown et al., 2021). Alternatively, person centered approaches such as cluster analysis and latent class/profile analysis, identify heterogeneous sub-groups of individuals who share similar attributes (Muthén & Muthén, 2000). Therefore, person centered approaches group participants together based on multiple behaviors, and are well suited to understanding the effects of multiple physical activity behaviors on mental health.

A growing body of literature has used latent profile analysis (LPA) to examine physical activity profiles and their effects on health outcomes. For example, Gupta et al. (2020) identified four unique profiles based on combination time use behaviors (i.e., sedentary, standing, light intensity physical activity, MVPA) during work and leisure-time and compared indicators of obesity between profiles. von Rosen et al. (2020) identified three unique profiles based on a combination of movement behaviors and compared odds of all-cause mortality at 15-years follow-up between profiles. Additionally, Wolvers et al. (2018) found three unique profiles based on MVPA and sedentary behaviors, and the distribution of these behaviors throughout the day in a sample of adult cancer-survivors. Research has shown that mental health and wellbeing may differ between older adults residing in assisted living facilities with unique movement and functioning profiles (Park et al., 2017). Participants in an active lifestyle and high physical functioning profile reported significantly more positive quality of life, vitality and mental wellbeing, significantly less symptoms of depression and anxiety, and less fatigue than participants in the inactive lifestyle and low physical functioning profile. However, to the best of the author’s knowledge, no studies have identified profiles based on aerobic and muscle strengthening physical activities, and examined differences in wellbeing and quality of life between profiles in adults from the general population.

The aims of this study are to (1) identify distinct pre-COVID-19 lockdown physical activity profiles (i.e., walking, leisure-time MVPA, domestic MVPA and muscle strengthening exercise) in a sample of adults from the general population and (2) assess whether identified profiles are associated with mental wellbeing and quality of life during COVID-19 lockdown.

## Methods

### Participants and procedures

Data for the current study was collected between May and August 2020 as baseline data for a planned larger study on the impact of a new health and well-being hub on residents’ PA and mental wellbeing in a community in Melbourne, Australia. Victoria University Human Research Ethics Committee approved the study. The study sought participation from adult residents living in a Local Government area located in the Western metropolitan region of Melbourne, recruited via advertisement through the municipal government services (e.g., leisure centers, libraries) and local resident-run community social media groups. Participation in the study was voluntary, and informed consent was obtained electronically or in paper-based form before completing a questionnaire. The online version of the questionnaire was available in English and Vietnamese, due to the large Vietnamese community residing in the area.

A total of 442 adult residents completed the questionnaire. On average, participants were aged 43.97 years (SD = 13.85) and 24.4% of participants identified as male. At the time of the study 13.6% were unemployed, 11.3% were retired, 46.4% considered their financial situation to be comfortable, 63.6% of participants were born in Australia, and almost all (82.4%) spoke English at home.

## Measures

### Demographics

Demographic information collected from participants included age, gender, postcode, education, country of birth, language spoken at home, employment status and financial circumstances.

## Physical activity

The Active Australia Survey (AIHW, 2003) was adapted to assess pre-COVID lockdown aerobic physical activity. The Active Australia Survey includes eight items to measure the number of times and total minutes participants engaged in walking continuously for at least 10 min, domestic MVPA (e.g., gardening or heavy yard work), moderate intensity leisure-time physical activity (e.g., gentle swimming), and vigorous intensity leisure-time physical activity (e.g., jogging) in the last week. The preface of items was modified to assess typical pre-COVID-19 lockdown PA habits (e.g., *“In a usual week prior to COVID-19 social distancing restrictions, how many times did you…”*). Minutes of leisure-time MVPA were calculated by combining moderate and vigorous-intensity physical activity in a usual week prior to COVID-19. Consistent with other studies, vigorous-intensity physical activity was not weighted when calculating MVPA (Curtis et al., 2020; Fjeldsoe et al., 2013).

Additionally, a single item from the National Health Survey (2017-18) (ABS, 2018) was used to measure the number of times participants engaged in muscle strengthening exercise in a usual week. Participants were asked, *“Some activities are designed to increase muscle strength and tone, such as lifting weights, pull-ups, push-ups or sit-ups. How many times did you do any strength or toning activities in a usual week [prior to COVID-19 distancing restrictions]?”*

## Mental wellbeing

Mental wellbeing was assessed using the Short Warwick-Edinburgh Mental Well-being Scale (SWEMWBS) (Tennant et al., 2007). The SWEMWBS is a 7-item, positively worded scale that asks participants to respond to how often they experience thoughts and feelings over the previous two weeks, (e.g., *“I’ve been feeling optimistic about the future”).* Responses are given on a 5-point Likert scale from 1 (none of the time) to 5 (all of the time). A total score was calculated from 7 to 35, with higher scores indicating more positive mental wellbeing. The Cronbach’s alpha for the SWEMWBS in the current study was 0.86 indicating good internal reliability.

## Health related quality of life

Health related quality of life was assessed using the Assessment of Quality of Life (AQoL) instrument (Hawthorne et al., 1999). The AQoL is a 12-item scale that measures several domains of health-related quality of life, including independent living, social relationship, physical senses, and psychological wellbeing. Each item is scored on a 4-point scale, and a total score between 12 and 48 was calculated. A lower score indicates better health related quality of life. The Cronbach’s alpha for the AQoL in the current study was 0.74 indicating acceptable internal reliability.

### Statistical analysis

Data was checked for missingness, and although only 7.87% of values were missing, data were not missing completely at random (Little’s MCAR = 225.034, p < .001). Therefore, missing values were imputed using single regression imputation in SPSS (v. 26). Single regression imputation was used instead of multiple imputations, as it is possible that a different number of profiles could be estimated for each of the multiple imputations.

To determine distinct physical activity profiles, LPA was conducted using Mplus (v, 8.4; Muthén & Muthén 2017). Before completing the LPA, the data was checked for multivariate outliers (Tabachnick & Fidell, 2007). A total of 17 outliers were identified and excluded from analysis. A series of models with an increasing number of profiles were estimated to determine the optimal number of profiles based on minutes of overall walking, minutes of leisure-time MVPA, minutes of domestic MVPA and number of sessions of muscle strengthening exercise in a usual week. The Bayesian Information Criterion (BIC; Schwarz, 1978) and sample size adjusted BIC were used to determine the optimal number of profiles, with a lower BIC indicating better model fit (Nylund et al., 2007). Additionally, the Lo-Mendell-Rubin likelihood test (LMRT; Lo et al., 2001) and the bootstrapped likelihood ratio test (BLRT; McLachlan 1987) was used to compare the model fit between a model with k profiles compared to a model with k -1 profiles, with statistical significance indicating better model fit for the model with more profiles. The entropy criterion was reported and indicates how well separated the profiles are in a model, with a value closer to 1 indicating clearer delineation between profiles (Celeux & Soromenho, 1996). Additionally, the probability of profile membership and the interpretability of the profiles was considered when deciding on the optimal number of profiles.

Once the ideal number of profiles were identified, the manual three-step approach (Asparouhov & Muthén, 2014) was utilized to determine differences in mental wellbeing and health related quality of life between profiles. Equality of means were estimated using Wald’s test. Unadjusted models and models that adjusted for covariates were both estimated.

## Results

### Latent profiles

The summary of model fit from the LPA models with 2–6 profiles is in Table 1. The BIC, the sample size adjusted BIC and BLRT suggest improving model fit up to 6-profiles. Although the 6- profile models had better model fit, it was determined that the 5-profile model had greater theoretical value and was, therefore, easier and more meaningful to interpret. As such, the 5-profile model was chosen.

**Table 1:** Model fit indices for latent profile analysis

	Profile 1	Profile 2	Profile 3	Profile 4	Profile 5
Unadjusted	23.76(0.31)^ab^	24.39(0.46)^c^	23.00(0.96)^de^	25.37(0.69)^ad^	26.85(0.86)^bce^
Adjusted *	24.53(70.70)^a^	24.53(0.70)	23.10(0.93)^bc^	25.33(0.89)^b^	26.38(0.96)^ac^

^abcde^ Values that share a subscript with each other in the same row were significantly different at p < .05

* Adjusted for age, gender, employment status, retirement status, financial status and country of birth

Summary statistics for overall walking, leisure-time MVPA, domestic MVPA, and muscle strengthening sessions per week for each profile are shown in Table 2. Profile 1 (n = 220, 51.5%) was characterized by moderate walking, low leisure-time and domestic MVPA and very low muscle strengthening exercise. Profile 2 (n = 129, 30.2%) was characterized by moderate-to-high walking, moderate leisure-time MVPA, moderate domestic MVPA, and sufficient muscle strengthening exercise. Profile 3 (n = 17, 4.0%) was characterized by moderate-to-high walking, moderate leisure-time MVPA, very high domestic MVPA and very low muscle strengthening exercise. Profile 4 (n = 37, 8.7%) was characterized by moderate-to-high walking and leisure-time MVPA, moderate domestic MVPA, and very high muscle strengthening exercise. Lastly, Profile 5 (n = 24, 5.6%), was characterized by very high walking and leisure-time MVPA, moderate-to-high domestic MVPA, and high muscle strengthening exercise.

**Table 2:** Summary statistics by latent profiles

Number of profiles	BIC	ssaBIC	LMRT	BLRT	Entropy	Lowest probability of profile membership
2	18064.093	18023.578	< 0.001	< 0.001	0.92	16.6%
3	18005.199	17948.078	0.051	< 0.001	0.93	3.0%
4	17954.648	17881.660	0.002	< 0.001	0.92	3.1%
5	17903.512	17814.657	0.196	< 0.001	0.94	4.0%
6	17862.083	17757.361	0.513	< 0.001	0.95	4.0%

## Comparison of wellbeing and health related quality of life by latent profiles

Differences in wellbeing between the profiles are shown in Table 3. The unadjusted model showed that participants in Profiles 4 and 5 reported significantly greater wellbeing than participants in Profile 1 and Profile 3. Additionally, participants in profile 5 reported significantly greater wellbeing than participants in Profile 2. When controlling for covariates, there was no longer a significant difference in wellbeing between Profile 1 and Profile 4, or between Profile 2 and Profile 5.

**Table 3:** Mental wellbeing by latent profiles

	Overall walking mins/week M(SE)	Leisure-time MVPA mins/week M(SE)	Domestic MVPA mins/week M(SE)	Muscle strengthening exercises sessions/week M(SE)
Profile 1	168.86(11.19)	89.97(8.88)	52.94(5.58)	0.16(0.03)
Profile 2	226.85(14.10)	193.83(15.49)	87.30(9.30)	2.47(0.08)
Profile 3	224.13(66.43)	183.50(52.29)	360.14(56.59)	0.38(0.06)
Profile 4	228.81(34.15)	256.09(37.02)	88.00(32.25)	6.02(0.19)
Profile 5	488.64(58.39)	608.56(56.99)	192.90(46.58)	3.77(0.28)

Differences in health related quality of life between the profiles are shown in Table 4. Results showed that participants in Profile 2 and Profile 5 reported significantly better health related quality of life than participants in Profile 1 in both adjusted and unadjusted models. There was no difference in health-related quality of life between any of the other profiles.

**Table 4:** Health related quality of life by latent profiles

	Profile 1	Profile 2	Profile 3	Profile 4	Profile 5
Unadjusted	18.03(0.28)^ab^	16.64(0.29)^a^	17.12(1.38)	17.36(0.71)	16.08(0.48)^b^
Adjusted *	18.25(0.47)^ab^	17.04(0.45)^a^	17.18(1.24)	17.69(0.77)	16.57(0.59)^b^

^abc^ Values that share a subscript with each other in the same row were significantly different at p < .05

* Adjusted for age, gender, employment status, retirement status, financial status and country of birth

## Discussion

This study identified distinct pre-COVID-19 physical activity profiles based on different types of self-reported physical activity in a sample of adults and assessed if any of these profiles served as a protective buffer for mental wellbeing and quality of life during COVID-19 lockdown.

Overall, five unique pre-COVID-19 physical activity profiles were identified that differed in terms of levels of walking, leisure-time MVPA, domestic MVPA and muscle strengthening exercise. Profiles ranged from moderate walking and low-to-moderate leisure-time MVPA and very little muscle strengthening exercise, to high-to-very high walking, leisure-time MVPA and muscle strengthening exercise. Additionally, a profile characterized by very high levels of domestic MVPA and a profile characterized by very high levels of muscle strengthening exercise with low to moderate levels for all other types of physical activity were identified. Just over half of all participants were assigned to the low-to-moderate MVPA and insufficient muscle strengthening exercise profile.

This study’s findings add to a previous LPA study that found three profiles in their sample: low, average and high physical activity (von Rosen et al., 2020). Like this past study, most participants in the current study were assigned to profiles characterized by low, moderate, and high levels of all physical activity indicators. There were far fewer participants in profiles that were highly active for a single type of physical activity, such as domestic MVPA or muscle strengthening exercise, and low-to-moderate levels for all other types of activities. This suggests that physical activity behaviors likely co-occur, and it is unlikely that an individual will be highly active in one type of physical activity and inactive in another. An exception may be for differences in leisure-time and occupational physical activity (not measured in the current study); previous studies have shown that people may be highly active at work and inactive in their leisure-time, or vise-versa (Gupta et al., 2020).

The results from the current study add to previous research that used cut-points to determine physical activity profiles based on aerobic and muscle strengthening exercise guidelines. Previous studies found that 74–81% of participants do not adhere to both aerobic and muscle strengthening exercise guidelines based on physical activity across all domains (De Cocker et al., 2020; Oftedal et al., 2019). This is higher than the 55.5% of participants in the current study who were assigned to profiles that did not achieve both aerobic and muscle strengthening exercise guidelines across domestic and leisure-time physical activity. In addition to the results from this study suggesting a higher portion of participants achieve aerobic and muscle strengthening exercise guidelines than previous studies using cut-points, the results also showed that participants who did achieve guidelines may be broken down into further profiles, varying from moderately to very highly active profiles. Therefore, results from the current study highlight that participants who achieve recommended guidelines may differ substantially in the minutes of physical activity they participate in and the benefits of using a person-centered approach to examine co-occurrence of different physical activity behaviors.

This study also examined differences in mental wellbeing and health related quality of life between the different pre-COVID physical activity profiles. Results showed that participants in the very high MVPA and very high muscle strengthening exercise profiles reported significantly higher positive wellbeing than participants in the profiles that did not achieve both aerobic MVPA and muscle strengthening exercise guidelines. Additionally, results showed that wellbeing did not differ between the moderately active profile that achieved aerobic MVPA and muscle strengthening exercise guidelines and the profiles that did not achieve both guidelines. This indicates that the relationship between physical activity and wellbeing may not be linear. It may be important to engage in high aerobic MVPA or muscle strengthening exercise beyond the minimum levels suggested by physical activity guidelines. This finding supports the recommendation from the most recent global physical activity guidelines that endorse participating in greater physical activity than the minimum recommended levels for additional health benefits (Bull et al., 2020).

The present study also showed that participants in the very high MVPA profile reported significantly better health related quality of life than participants in the low-to-moderate MVPA and very low muscle strengthening exercise profile. Additionally, participants in the moderate MVPA and sufficient muscle strengthening exercise profile also reported significantly greater health related quality of life than participants in the low-to-moderate MVPA and very low muscle strengthening exercise profile. This is consistent with previous LPA research, which found that older adults in the more favorable physical activity and physical functioning profiles reported better quality of life than participants in less favorable profiles (Park et al., 2017). This also adds to research that has consistently shown a positive association between participation in aerobic physical activity and health related quality of life (Bize et al., 2007; Jantunen et al., 2019; Koolhaas et al., 2018).

Based on the observed profiles related to pre-COVID-19 lockdown physical activity participation, it appears that regular high levels of MVPA and muscle strengthening exercise may serve as a protective factor against the stressors of a global pandemic lockdown. Similar conclusions were drawn in a study assessing the mental health and movement patterns of 214 university students in the UK across time during the COVID-19 lockdown period (Savage et al., 2020). There were no associations between changes in physical activity and mental wellbeing, leading the authors to suggest that reductions in physical activity during lockdown were possibly not enough to adversely impact mental health. In line with this study’s findings, physical activity participation may be a protective buffer for mental health in periods of extreme disruption and uncertainty, as in the COVID-19 pandemic.

Interestingly, the current study found that participants who engaged in very high levels of domestic MVPA pre-COVID reported less positive wellbeing during the COVID-19 lockdown than participants who reported participating in higher levels of leisure-time MVPA or high levels of muscle strengthening exercise. The findings from the current study further highlight that the association between physical activity and mental health is likely domain specific (White et al., 2017b), and the importance of encouraging leisure-time physical activity to promote mental health (Teychenne et al., 2020).

The current study has several strengths, including using a person-centered method to examine regular physical activity as protective factor for mental wellbeing and quality of life during the COVID-19 lockdown period. This work has important implications for how the relationship between physical activity and mental wellbeing is understood as it enables simultaneous consideration of physical activity type, domain and dose in the association between physical activity behaviors and outcomes. These findings speak to the WHO’s (2020) conclusions that whilst physical activity type, domain, and dose are independently important factors in the association between physical activity and health-related outcomes, further exploration of how these specifically relate to optimal health benefits in different populations is necessary.

Regular physical activity has health benefits, including prevention of physical and mental ill health. However, the longer-term protective benefits for mental wellbeing during a rare global pandemic are not well understood. This study provides evidence that higher physical activity participation across types and domains are a protective mechanism for mental wellbeing and quality of life during a global pandemic where strict lockdown measures were imposed, impacting lives and livelihoods.

This study is not without limitations. As this study was conducted as part of planned larger study on the impact of a new health and well-being hub on residents’ physical activity and mental wellbeing in a local Melbourne community, recruitment methods may have led to an uncharacteristically active sample (e.g. advertising at leisure centres). This may explain why there were more adults in this study meeting physical activity guidelines than expected from previous literature. In addition, pre-COVID-19 physical activity data was collected retrospectively from participants and may be subject to recall error. Lastly, as this study explored physical activity as a protective factor, changes in physical activity participation due to COVID-19 lockdown restrictions were not considered in the analysis. However, it should be noted the changes in physical activity during lockdown might have affected wellbeing and quality of life.

In summary, the mental wellbeing and quality of life of people with high levels of physical activity before COVID-19 faired best during the pandemic strict lockdown period. Minimal prior physical activity levels are associated with the poorest health and wellbeing outcomes. However, the physical activity levels of individuals who achieve guideline-recommended physical activity levels vary widely, as do the associated health and wellbeing outcomes. Future studies should continue to utilize person-centered methods to explore physical activity related patterns and outcomes to understand better the effects of multiple physical activity types, domains and doses on health and wellbeing.

## Data Availability

Data is not available to share due to ethical constraints and for privacy purposes.
